# Effects of selenomethionine on intestinal microbiota and its metabolism in mice infected with porcine deltacoronavirus

**DOI:** 10.3389/fmicb.2025.1632166

**Published:** 2025-09-05

**Authors:** Haiyan Li, Yaya Shi, Tongjun Zhang

**Affiliations:** ^1^School of Physical Education, Yan'an University, Yan'an, Shaanxi, China; ^2^Division of Science and Technology, Yan'an University, Yan'an, Shaanxi, China

**Keywords:** PDCoV, SeMet, mice, intestinal microbiota, metabolomics

## Abstract

**Introduction:**

Porcine deltacoronavirus (PDCoV) is a novel enteropathogenic porcine coronavirus that primarily affects the intestinal tract. Modulating the intestinal microbiota could alleviate clinical signs and maintain the physical and chemical barrier of the intestines in piglets infected with PDCoV. Our previous study showed that selenomethionine (SeMet) could attenuate intestinal damage in PDCoV-infected piglets or mice. However, its influence on gut microbiota and metabolites is still unclear. We aimed to investigate the effect of SeMet on gut microbiota and metabolites in PDCoV-infected mice.

**Methods:**

In this study, samples of the contents of the colon were collected from mice in the Control group, the PDCoV group, and the SeMet+PDCoV group (0.3 mg/kg Se). These samples were analyzed using 16S rRNA sequencing, metabolomics analysis, and bioinformatics software to investigate the correlation between the gut microbiota and metabolites.

**Results:**

This study suggested that PDCoV infection could induce disorder in the intestinal microbiota of mice. SeMet treatment was found to restore the balance of this microbiota, including the bacteria Lactobacillus and Bifidobacterium. Altered intestinal microbiota also affect gut metabolism. Supplementing with SeMet brought the metabolites of the intestinal microbiota of PDCoV-infected mice closer to those of the Control group. These metabolites included phenylalanine-proline, tyrosine-proline, tyrosine, tryptophan, glutamate and octadecanamide. This contributed to an improved antiviral infection and immune response. Correlation analysis revealed a strong correlation between the gut microbiota and its metabolites.

**Conclusion:**

The alleviating effect of SeMet on intestinal damage caused by porcine delta coronavirus may be related to the intestinal microbiota and its metabolites.

## 1 Introduction

Porcine deltacoronavirus (PDCoV) is a novel porcine enteropathogenic coronavirus that has been shown to be transmissible between species and can cause intestinal damage in pigs, chickens, and mice (Li et al., 2020a,b). More recently, PDCoV was detected and isolated from three children in Haiti who presented with acute febrile illness (Lednicky et al., 2021), highlighting the serious threat of PDCoV to human health. Some broad-spectrum antivirals, such as lithium chloride, diammonium glycyrrhizinate and remdesivir, are being investigated for their potential to treat PDCoV *in vitro* (Zhai et al., 2019; Brown et al., 2019). It has also been found that remdesivir has a relatively high therapeutic effect on PDCoV *in vitro* (Brown et al., 2019). There is no best choice for the prevention and treatment of PDCoV in animal models.

Selenium (Se) is an important micronutrient. It has many biological functions, including antioxidant, antiviral and immunomodulatory effects (Roman et al., 2014). There is increasing evidence that Se plays a chemopreventive role in cancer risk and incidence (Roman et al., 2014; Wallenberg et al., 2014) and viral diseases (Sepúlveda et al., 2002; Li et al., 2023; Sartori et al., 2016; Wang et al., 2020). It may play a chemopreventive role against cancer by scavenging reactive oxygen species (ROS), thereby preventing DNA damage and the onset of mutations (Selenius et al., 2010). Studies have shown that selenomethionine (SeMet) can inhibit PDCoV replication in LLC-PK cells in a dose-dependent manner (Ren et al., 2022). Our previous experiment showed that SeMet could alleviate the intestinal injury induced by PDCoV-infected piglets or mice (Li, 2021; Li et al., 2025). However, the possible mechanisms remain poorly understood.

In recent years, accumulating evidence has supported the idea that the gut microbiota plays an important role in the pathogenesis of PDCoV (Li et al., 2020a,b; Zhang et al., 2025; Zhang Y. et al., 2024). PDCoV infection induces an imbalance in the gut microbiota, reducing bacterial diversity (Li et al., 2020a), altering metabolites, and inducing inflammatory responses (Li et al., 2020b). Thus, gut barrier damage induced by PDCoV-infected piglets can be attenuated by modulating the gut microbiota (Zhang et al., 2025). Our previous studies have shown that FMT can alleviate the clinical signs of PDCoV infection in piglets by modulating the intestinal flora and the composition of the intestinal barrier, thereby reducing the inflammatory response (Zhang et al., 2025). In addition, host Se levels can also influence the composition of the intestinal microbiota and the colonization of the gastrointestinal tract, thereby affecting selenium status (Kasaikina et al., 2011; Ferreira et al., 2021). Feeding Se-enriched supplements to mice can regulate the gut microbiota and host metabolism and reduce intestinal inflammation in mice (Wang H. et al., 2024). However, the potential effect of SeMet on the gut microbiota and its metabolites in PDCoV-infected mice has not been investigated.

The current study used C57BL/6 (C57) mice as a model of PDCoV infection to explore the effect of SeMet on intestinal microbiota and its metabolites in mice infected with PDCoV.

## 2 Materials and methods

### 2.1 Virus and animals

The virulent PDCoV HNZK-02-P5 strain was provided by Professor Zhanyong Wei in Henan Agricultural University.

Four-week-old C57 mice (female, approximately 16 g) were randomly divided into 3 groups (10 mice per group), namely Control group, PDCoV group, and SeMet+PDCoV group. These mice were supplemented with SeMet and subjected to viral infection experiments as previously described (Li et al., 2025). The mice in the SeMet + PDCoV group were fed with a SeMet-treated diet (0.3 mg/kg Se) daily from day 1 until the end of the experiment. On day 23, the mice in the PDCoV group and the SeMet+PDCoV group were inoculated intragastrically with 300 μL per mouse of PDCoV HNZK-02-P5 strain (1 × 10^6^ TCID_50_), and the mice in the Control group were inoculated with the same volume of Dulbecco's modified Eagle's medium (DMEM). On day 28, all mice in the experimental groups were necropsied. The intestinal tissues were collected to measure viral load and conduct histopathological analysis. The colonic contents were collected and stored at −80 °C until high-throughput sequencing (Illumina MiSeq) of the 16S rRNA gene and LC-MS/MS metabolomics were assessed. All operations were approved by the Medical Ethics Committee of Yan'an University Affiliated Hospital (Yan'an, China).

### 2.2 DNA extraction from the colonic content and 16S rRNA sequencing

To analyse the composition of the microbial community, 16S rRNA sequencing was performed on samples of the mouse colon. The FastDNA^®^SPIN Kit (MP Bio, United States) was used to extract microbial DNA according to the manufacturer's instructions. DNA concentration was assessed using a NanoDrop 2,000 UV-vis spectrophotometer (Thermo Scientific, Wilmington, USA). The integrity of genomic DNA was assessed by 1.0% agarose gel electrophoresis. All DNA was stored at −20 °C until further analysis.

For 16S rRNA sequencing, the hypervariable regions V3-V4 of the 16S rRNA gene were amplified using universal primers 338F and 806R (Mori et al., 2013). The products were sequenced on the Illumina MiSeq (Illumina, San Diego) and the sequences were analyzed using standard protocols provided by Majorbio Bio-Pharm Technology Co. Ltd. (Shanghai, China).

### 2.3 Metabolomic signatures

UHPLC-Q Exactive HF-X technology was used to analyse the metabolic profiles of the control, PDCoV, and SeMet groups. A thawed sample (50 mg) was accurately weighed and immediately ground to powder.

The sample was homogenized in 1 mL of pre-cooled methanol/ddH_2_O solvent (4:1, v/v). The mixture was treated with cryogenic ultrasound at 5 °C for 30 min, placed at −20 °C for 30 min and then centrifuged at 4 °C and 13,000 g for 15 min. The supernatant was extracted and lyophilised using a vacuum lyophiliser, and the dried sample was redissolved in 100 μL acetonitrile/ddH_2_O solvent (1:1, v/v) for homogenisation. After centrifugation at 4 °C and 14,000 rpm for 15 min, the supernatant was collected for subsequent analyses. Additional quality control (QC) samples were prepared to monitor instrument stability and repeatability.

The UHPLC-Q Exactive HF-X system was analyzed for LC-MS/MS using the following LC conditions: Mobile phase A, 0.1% formic acid in water: acetonitrile (95:5, v/v), mobile phase B, 0.1% formic acid in acetonitrile: isopropanol: water (47.5:47.5, v/v), flow rate, 0.4 mL/min. MS conditions were set as follows: Positive and negative ion scan mode is used to collect the sample quality spectrum signal, and the quality scan range is m/z: 70–1,050. The floating ion spray voltage was 3,500 V and 2,800 V for positive and negative modes. The sheath gas, auxiliary heating gas, and ion source heating temperature were 40 psi, 10 psi, and 400 °C respectively. The cyclic collision energy, MS1 resolution and MS2 resolution were 20-40-60 V, 70,000 and 17,500 respectively.

### 2.4 Combined different species and metabolome analyses

A comprehensive analysis was conducted to examine both different species and differentially accumulated metabolites. Pearson's correlation analysis was performed for different species and metabolites detected in the Control, PDCoV, and SeMet + PDCoV groups by Euclidean distance.

### 2.5 Bioinformatic analysis of the sequencing data

Bioinformatics, statistical, and visual analyses were performed using QIIME and the R package (v3.1.1). Sequences were clustered into operational taxonomic units (OTUs) using UPARSE (version 3.3.1) with a 97% similarity cut-off and used for further analysis of rarefaction curves, Venn diagrams, and alpha diversity indices (Shannon, Simpson, ACE, Chao, and Good's coverage) using Mothur software v1.30.1. Beta diversity was based on weighted UniFrac distance analysis. At the phylum, family, and genus levels, community bar plots were used to show the structural composition of communities in different subgroups.

Metabolite data processing was performed using Progenesis QI software (Waters Corporation, Milford, USA). Simultaneously, metabolites were identified by database searches, the main databases being HMDB, Metlin, and MajorBio Database. Based on the variable importance in projection (VIP) obtained by the OPLS-DA model, the metabolites with VIP > 1 and *P* < 0.05 were determined as significantly different metabolites, with the *p*-value generated by Student's *t*-test. Differential metabolites were mapped to their biochemical pathways using pathway and enrichment analysis based on the KEGG database (http://www.genome.jp/kegg/). Spearman correlation analysis was performed on the different species and metabolites obtained by difference analysis.

### 2.6 Statistical analysis

One-way analysis of variance (ANOVA) was performed to analyse the significance between groups, and the Tukey *post hoc* test was used for multiple comparisons. GraphPad Prism 6.0 was used for statistical tests and graphs. Results are presented as mean ± standard deviation (SD). Statistical details are provided in the figure legends. *P* < 0.05 was considered statistically significant, ^*^, *P* < 0.05; ^**^, *P* < 0.01.

## 3 Results

### 3.1 SeMet regulated intestinal microbiota in mice infected with PDCoV

#### 3.1.1 Sequencing data quality analysis

**Figure 1:**
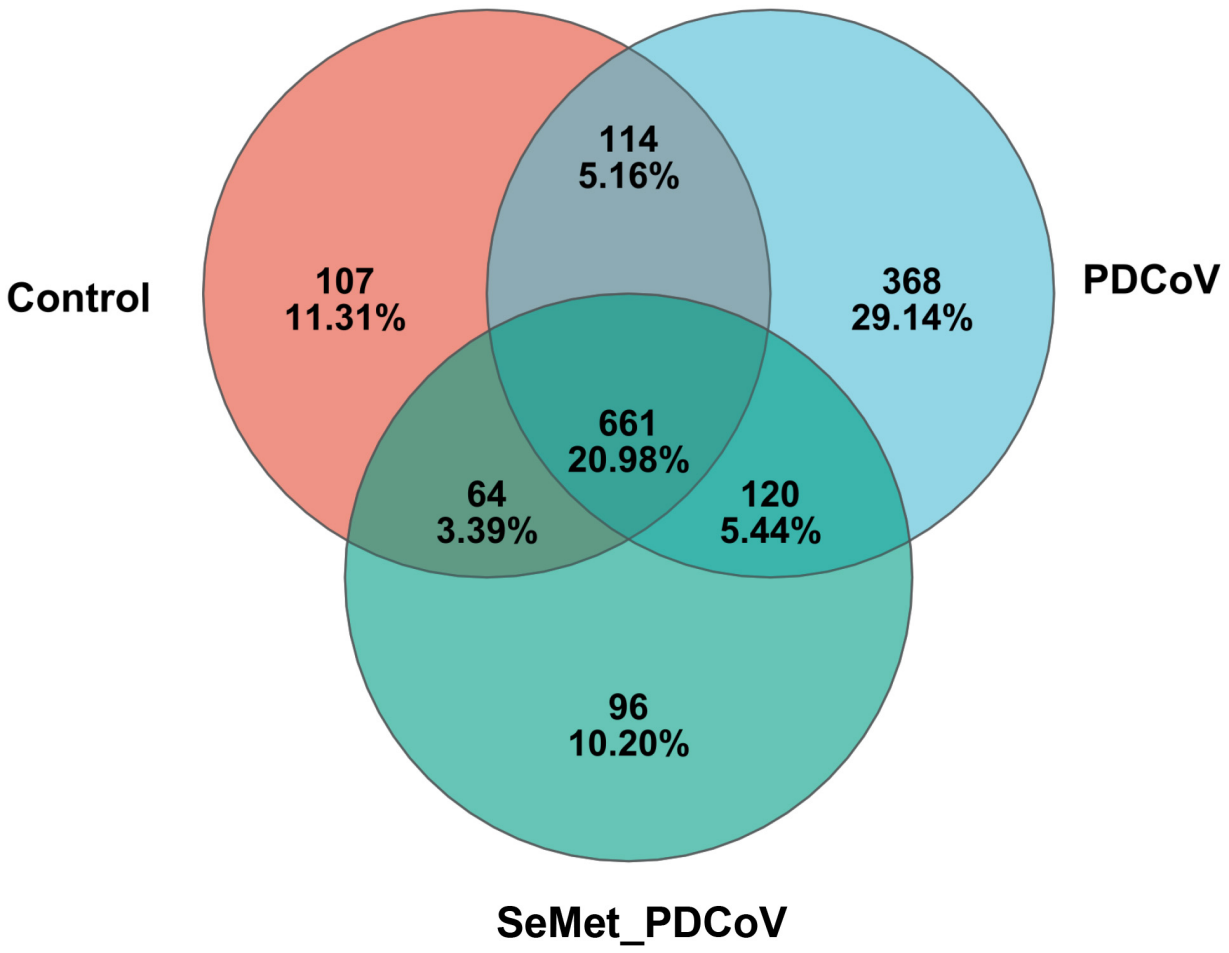
Venn diagram of different groups. Venn map of shared OTUs based on the sequences with more than 97% similarity (*n* = 10). a Venn diagram displaying the shared or unique OTU sequences among the control group, the PDCoV group, and the SeMet + PDCoV group was developed. The SeMet_PDCoV in the figure represents the SeMet+PDCoV group (The same as the following figures).

Thirty intestinal samples from three treatment groups were analyzed by 16S rRNA gene sequencing. A total of 1,760,944 valid and high-quality sequences were collected, with a median length of 417 bp OTUs. The average coverage of each sample was more than 99%, and more than 80% of the sequences in the samples passed the quality test, suggesting that the results of this sequencing may reflect the true situation of the gut flora in the samples. Based on the Greengenes database using QIIME, OTUs were generated, and those with more than 97% similarity were categorized (including phylum, family, and genus). The Venn diagram showed that there were 946 OTUs in the Control group, 1,263 OTUs in the PDCoV group, and 941 OTUs in the SeMet-PDCoV group. There were 661 OTUs common to all sequenced samples (Figure 1).

#### 3.1.2 Analysis of the alpha diversity and characterization index of intestinal flora

**Figure 2:**
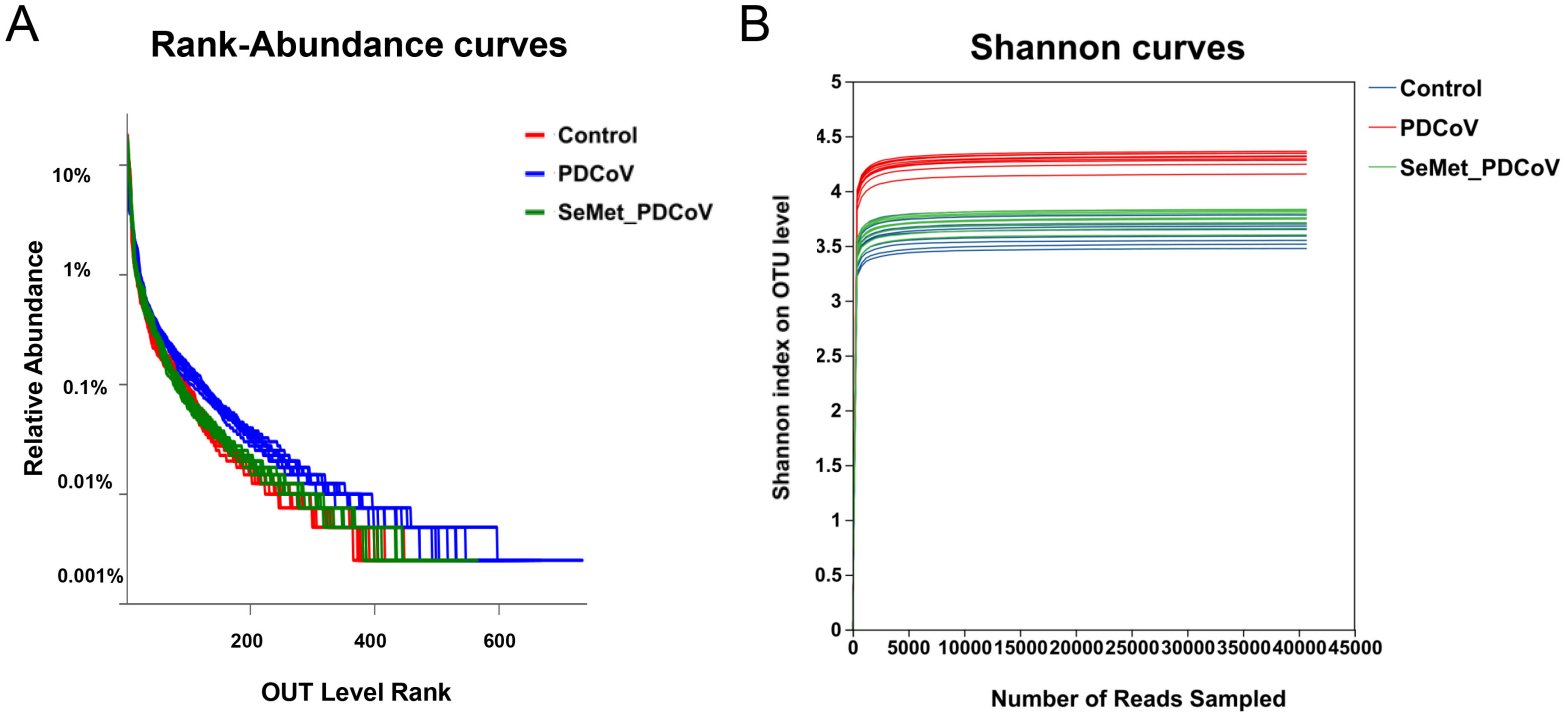
The OTU Rank-abundance curve and Shannon index curve of colonic microbiota of mice. **(A)** OTU Rank-abundance curve, curve width represents the richness of the community in the sample, flat curve represents the evenness of the community in the sample. **(B)** Shannon index curve, flat curve indicates sufficient sequencing data.

In order to further demonstrate the distribution pattern of species abundance, the OTU abundance rank curves and Shannon index curves were analyzed (Figure 2). The results showed that the OTU Rank curves all had a long “tail” (Figure 2A), implying that most of the bacterial species in the colonic flora of the mice in this experiment were gradually distributed evenly. The range that the curves spanned on the horizontal axis indicated that most bacterial species were more abundant. The curves in Figure 2B are all flat, indicating that the amount of sequencing data is sufficient to reflect the majority of the microbial diversity information of the samples in this experiment.

Under the condition that the sample size and sequencing depth are qualified, OTUs and the species richness (ACE and Chao) and diversity (Shannon and Simpson) from each sample were evaluated (Table 1). A closer inspection of Table 1 showed that the species richness index (ACE and Chao) and diversity index (Shannon) of the PDCoV group showed an increasing tendency compared to that in the Control and SeMet+ PDCoV groups, but none of these differences was statistically significant (*P* > 0.05).

**Table 1:** Effects of SeMet on intestinal microbial species alpha diversity in PDCoV-infected mice.

**Group**	**No. of sequences**	**No. of OTUs**	**Coverage (%)**	**Richness estimator**		**Diversity index**	
				**ACE**	**Chao**	**Shannon**	**Simpson**
Control	5,54,764	946	99.94	172.88 ± 11.88	172.43 ± 13.87	2.89 ± 0.05	0.10 ± 0.01
PDCoV	6,15,857	1,263	99.95	175.92 ± 4.20	176.40 ± 15.58	2.98 ± 0.06	0.10 ± 0.01
SeMet + PDCoV	5,90,323	941	99.95	174.30 ± 8.82	173.48 ± 8.13	3.00 ± 0.07	0.08 ± 0.01

Alpha diversity values are represented as mean ± SD (*n* = 10). In the same row, values with no letter superscripts mean no significant difference (*P* > 0.05).

#### 3.1.3 Analysis of the beta diversity and characterization index of intestinal flora

**Figure 3:**
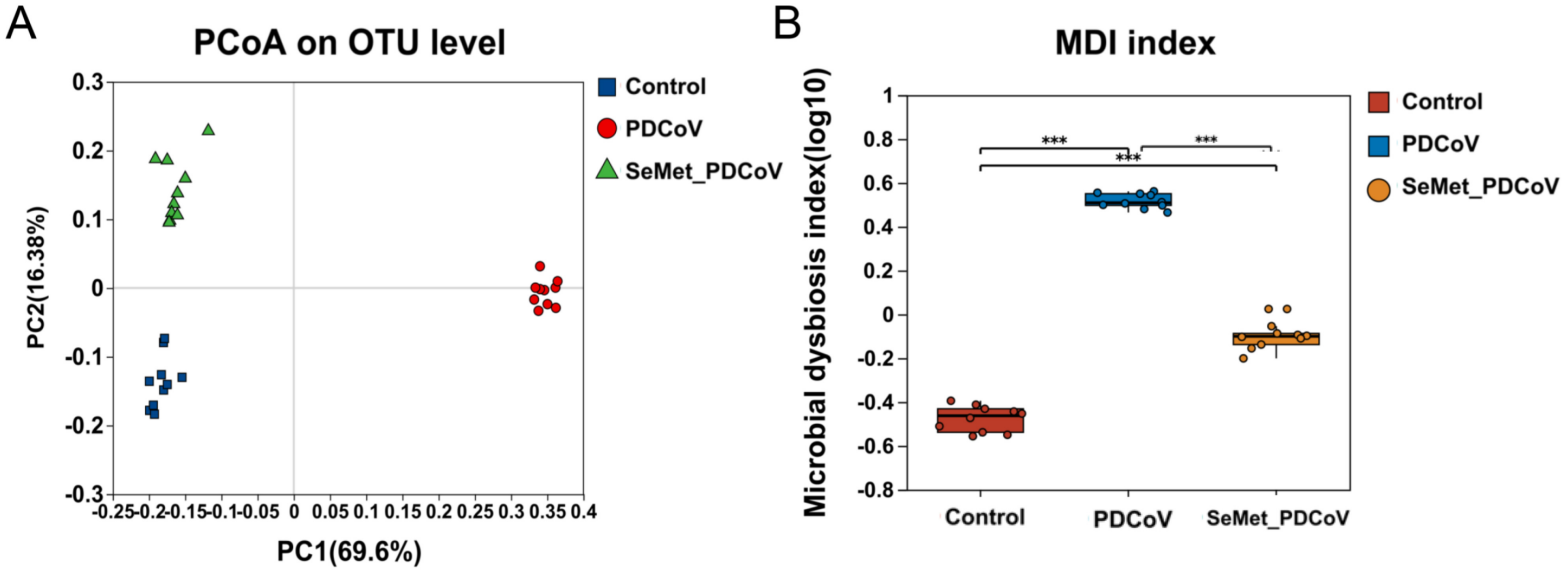
Effects of SeMet on intestinal microbial species Beta diversity and characterization index measures in PDCoV-infected mice. **(A)** PCoA analysis for the β-diversity of intestinal microbiota of the control group (blue), PDCoV group (red), and SeMet + PDCoV group (green). **(B)** Wilcoxon rank-sum test for the dysregulation index of intestinal microbiota of the control group (red), the PDCoV group (blue), and the SeMet + PDCoV group (yellow). (*n* = 10). MDI: microbial dysregulation index (***P < 0.001).

The PCoA based on Bray-Curtis distance metrics was performed to measure the similarity of colonic bacterial composition among the Control, PDCoV, and SeMet+PDCoV groups. The results showed a clear separation for the three groups, indicating a significant difference in colonic microbial structure (Figure 3A). Furthermore, the PDCoV group samples were tightly clustered. This difference in clustering suggests that microbiome perturbations are induced by PDCoV infection.

From the intestinal flora characterization index (Figure 3B), we found that PDCoV infection led to a significant increase in the microbial dysregulation index (MDI) (*P* < 0.001). The MDI in the SeMet + PDCoV group was significantly lower than in the PDCoV group, indicating that SeMet supplementation could improve intestinal flora disturbance induced by PDCoV infection in mice.

#### 3.1.4 Taxonomic composition analysis

**Figure 4:**
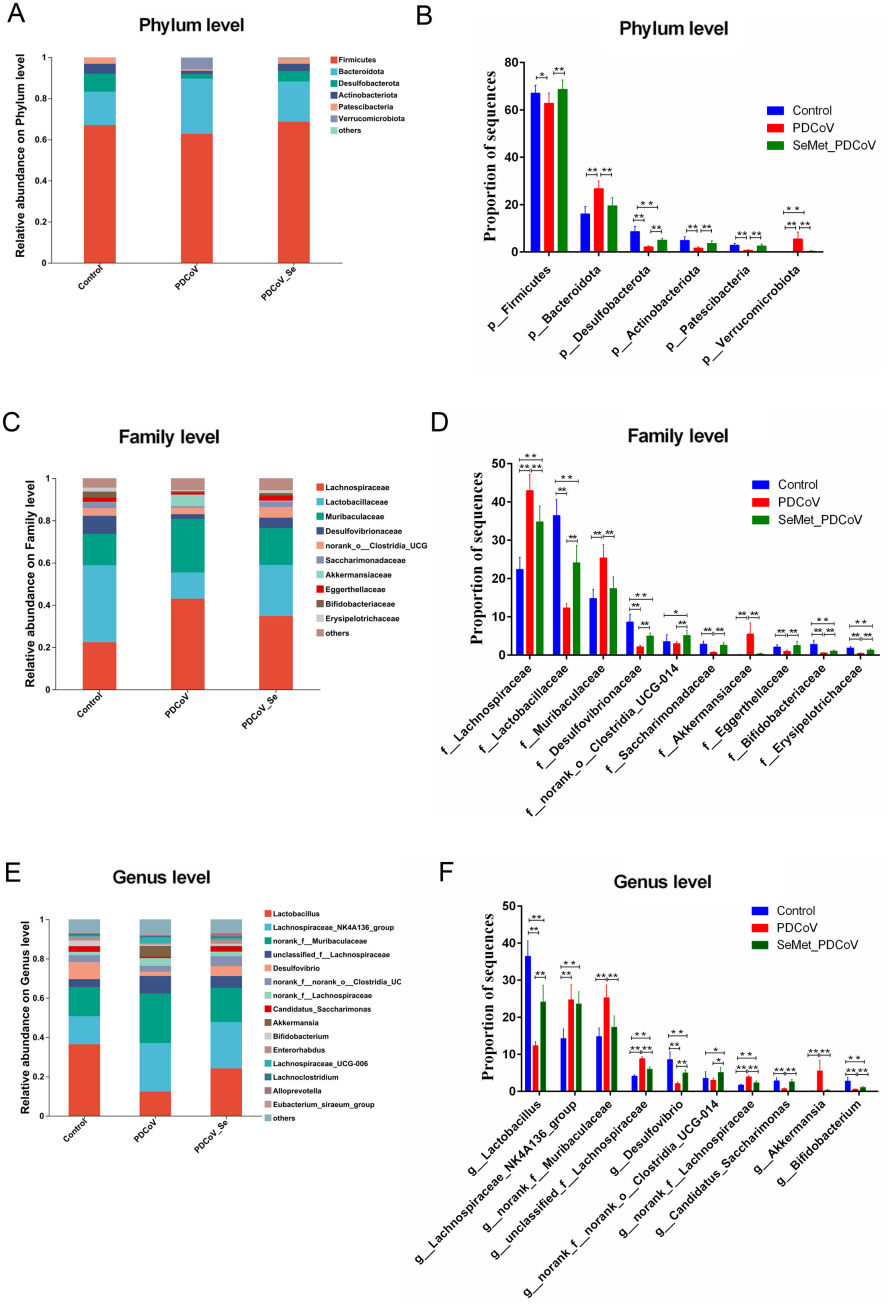
The effects of SeMet on the composition of bacterial taxa in PDCoV-infected mice. Relative abundances at the phylum **(A, B)**, family **(C, D)**, and genus **(E, F)** levels for colonic bacteria. Asterisks indicate a significant difference from the corresponding comparison group, (*n* = 10, *P < 0.05, **P < 0.01).

Next, we compared the relative abundance features of each group at the phylum, family, and genus levels to distinguish specific alterations in microbiota. As shown in Figures 4A–F, six phyla had the highest mean relative abundance, including the Firmicutes, Bacteroidota, Desulfobacterota, Actinobacteriota, Patescibacteria, and Verrucomicrobiota, and the proportion of the Firmicutes (*P* < 0.05), Desulfobacterota (*P* < 0.01), Actinobacteriota (*P* < 0.01), and Patescibacteria (*P* < 0.01) in the PDCoV group was lower than in the Control group. Still, the Bacteroidota and Verrucomicrobiota in the PDCoV group were higher than those in the Control group (*P* < 0.01). Interestingly, the supplementation of SeMet increased the proportion of the phyla Firmicutes, Desulfobacterota, Actinobacteriota and Patescibacteria (*P* < 0.01), whereas it decreased the phyla Bacteroidota, and Verrucomicrobiota (*P* < 0.01) compared to the PDCoV group (Figures 4A, B). In addition, compared with the control group, there was no significant difference in the proportion of Firmicutes and Bacteroides in the SeMet group (*P* > 0.05).

At the family level, the proportion of Lachnospiraceae, Muribaculaceae and Akkermansiaceae in the PDCoV group significantly increased compared with the control group (*P* < 0.01). Similarly, the proportion of Lactobacillaceae, Desulfovibrionaceae, norank_o_Clostridia UCG-014, Saccharimonadaceae, Eggerthellaceae, Bifidobacteriaceae, and Erysipelotrichaceae in the PDCoV group significantly decreased (*P* < 0.01). SeMet supplementation significantly reversed these changes (*P* < 0.01) (Figures 4C, D). There was no significant difference in the proportion of Muribaculaceae, Akkermansiaceae, Saccharimonadaceae, and Eggerthellaceae between the SeMet group and the control group (*P* > 0.05). At the genus level, the abundance of *Lactobacillus, Desulfovibrio, Candidatus_Saccharimonas*, and *Bifidobacterium* significantly decreased (*P* < 0.01). In contrast, the abundance of *Lachnosplraceae NK4A136_group, norank_f_Muribaculaceae, unclassified_f_Lachnospiraceae, norank_f_Lachnospiraceae*, and *Akkermansia* significantly increased in the PDCoV group (*P* < 0.01) As expected, SeMet supplementation significantly reversed these changes (*P* < 0.01), except for *Lachnosplraceae NK4A136_group* (*P* > 0.05) (Figures 4E, F). Overall, the results of the species difference analysis showed that SeMet supplementation could significantly regulate changes to the intestinal microbiota of mice induced by PDCoV.

### 3.2 SeMet regulated intestinal metabolic profile in mice infected with PDCoV

#### 3.2.1 Metabolite content change

**Figure 5:**
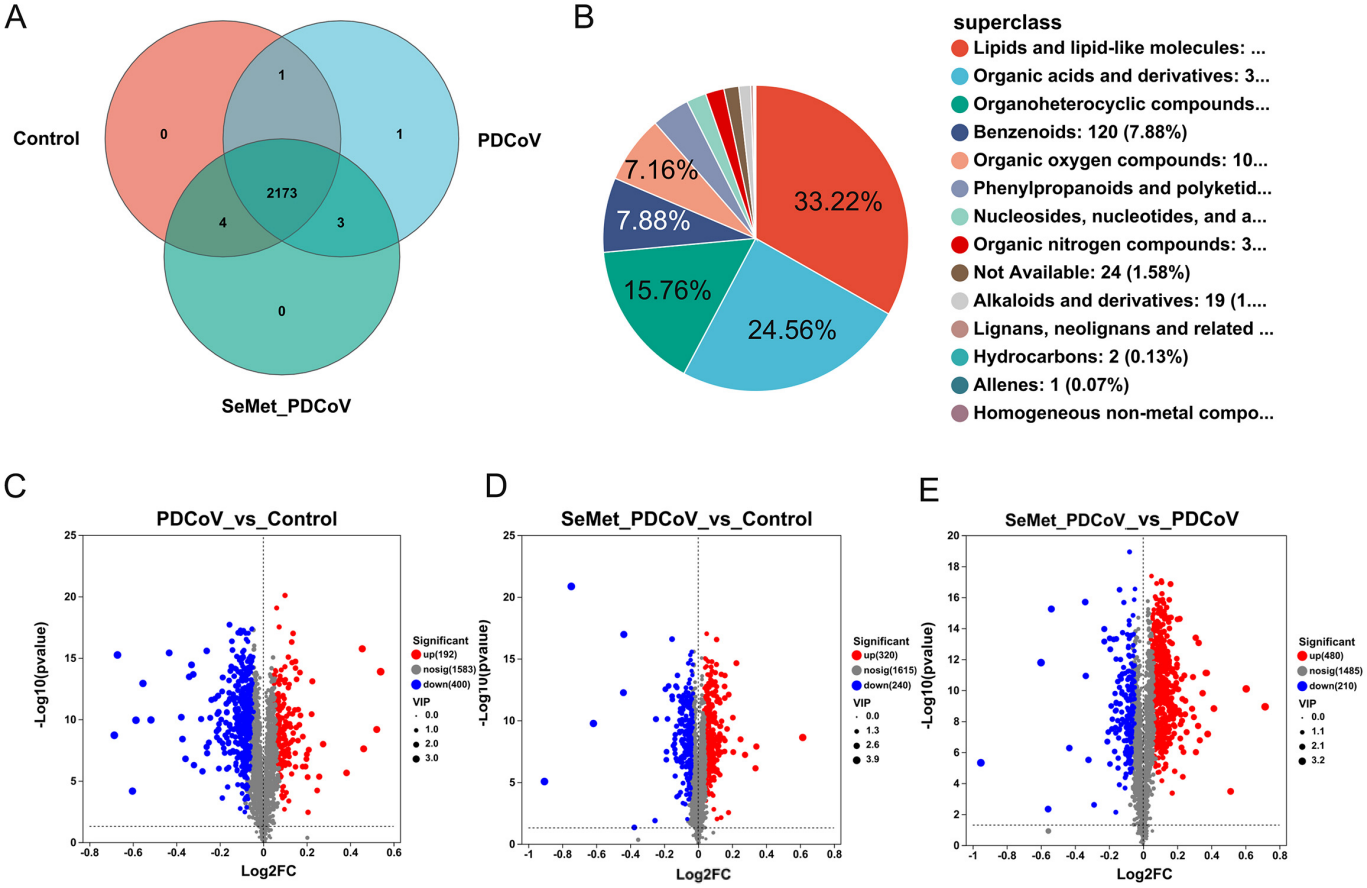
Differential metabolites among groups. **(A)** Venn diagram analysis among the Control, PDCoV, and SeMet + PDCoV groups, **(B)** Compound classification analysis among the Control, PDCoV, and SeMet + PDCoV groups, **(C)** Volcano plot of the PDCoV group vs. the Control group, **(D)** Volcano plot of the SeMet + PDCoV group vs. the Control group, **(E)** Volcano plot of the SeMet+PDCoV group vs. the PDCoV group (*n* = 6). Each volcano plot shows the differentially expressed genes between two groups. The x-axis shows the log_2_ scale of the fold change (log2FC) in gene expression. Positive values indicate an increase, while negative values indicate a decrease. The y-axis is the negative log10 scale of the adjusted p-value [-log10 (*p*-value)], which indicates the level of significance of the expression difference. Red dots represent significantly up-regulated genes, blue dots represent significantly down-regulated genes and gray dots represent genes with a non-significant difference.

To further determine the effect of SeMet on PDCoV-infected mice, an untargeted metabolomics study was performed on the collected colon contents. MetaboAnalyst analyzed the metabolite profiles of the colon contents. Identifiable metabolites were analyzed by Venn Diagram to calculate the number of common and unique metabolites in different groups (Figure 5A). The Venn diagram showed 2,178 OTUs in the control group, 2,177 OTUs in the PDCoV group, and 2,180 OTUs in the SeMet+PDCoV group. A total of 2,173 OTUs were shared among all sequenced samples.

In the present study, the intestinal contents of mice from all groups were analyzed for compound classification. Among them, lipids and lipoid molecules accounted for 33.22% of the metabolites, organic acids and their derivatives accounted for 24.56% of the metabolites, and organic heterocyclic compounds, benzene compounds, and organic oxygen compounds accounted for 20.8% (Figure 5B).

Volcano plots (*P*-value < 0.05 and fold change > 2) were used to identify significantly changed metabolites after treatment (Figures 5C, E). A total of 2,175 differential metabolites were detected between the PDCoV and control groups, of which 192 genes were upregulated and 400 were downregulated (Figure 5C). Following SeMet intervention, 480 genes were upregulated and 210 genes were downregulated in the gut metabolites of infected mice (Figure 5E). Meanwhile, 320 genes were upregulated and 240 genes were downregulated among the 2,175 differential metabolites compared to the control group (Figure 5D). These results suggest that changes in the gut microbiota led to significant changes in metabolites and that SeMet also regulated the metabolite changes induced by PDCoV infection in mice.

#### 3.2.2 Cluster analysis of metabolites

**Figure 6:**
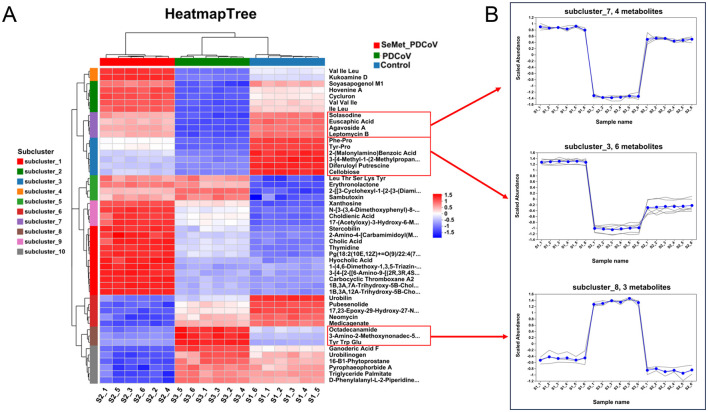
The effects of SeMet on the cluster of differential metabolites in mice infected with PDCoV. **(A)** Clustered heatmap analysis among the Control, PDCoV, and SeMet + PDCoV groups. **(B)** Subcluster trend graph analysis among the Control, PDCoV, and SeMet + PDCoV groups (*n* = 6).

Cluster analysis is a valuable method for identifying trends of differential metabolites in different groups. Therefore, we performed a cluster heat map analysis of a total of 541 differential metabolites in 3 groups. The results showed that there were ten significantly enriched gene clusters (Figure 6A). To visually illustrate the differences in metabolites among groups, subcluster trend graphs were generated. An interesting phenomenon is found in subclusters 3, 7, and 8 (Figure 6B). In subclusters 3 and 7, PDCoV down-regulated 10 metabolites, mainly involved in lipids and lipid-like molecules, organic acids and derivatives, organoheterocyclic compounds, benzenoids, and other compounds, including phenylalanine (Phe)-proline (Pro), tyrosine (Tyr) and proline (Pro), 2-(Malonylamino) benzoic acid, 3-[4-methyl-1-(2-methylpropyl)] benzoic acid, diferuloylputrescine, cellobioside, solasodine, euscaphic acid, agavoside A, and leptomycin B. Meanwhile, in subcluster 8, PDCoV up-regulated octadecanamide, 3-amino-2-methoxynonadec-5-en-4-one, and tyrosine, tryptophan, and glutamate (Figure 6B). Conversely, SeMet reversed these changes, which may explain why SeMet protects against PDCoV infection in mice.

#### 3.2.3 Metabolic pathway and network analysis

**Figure 7:**
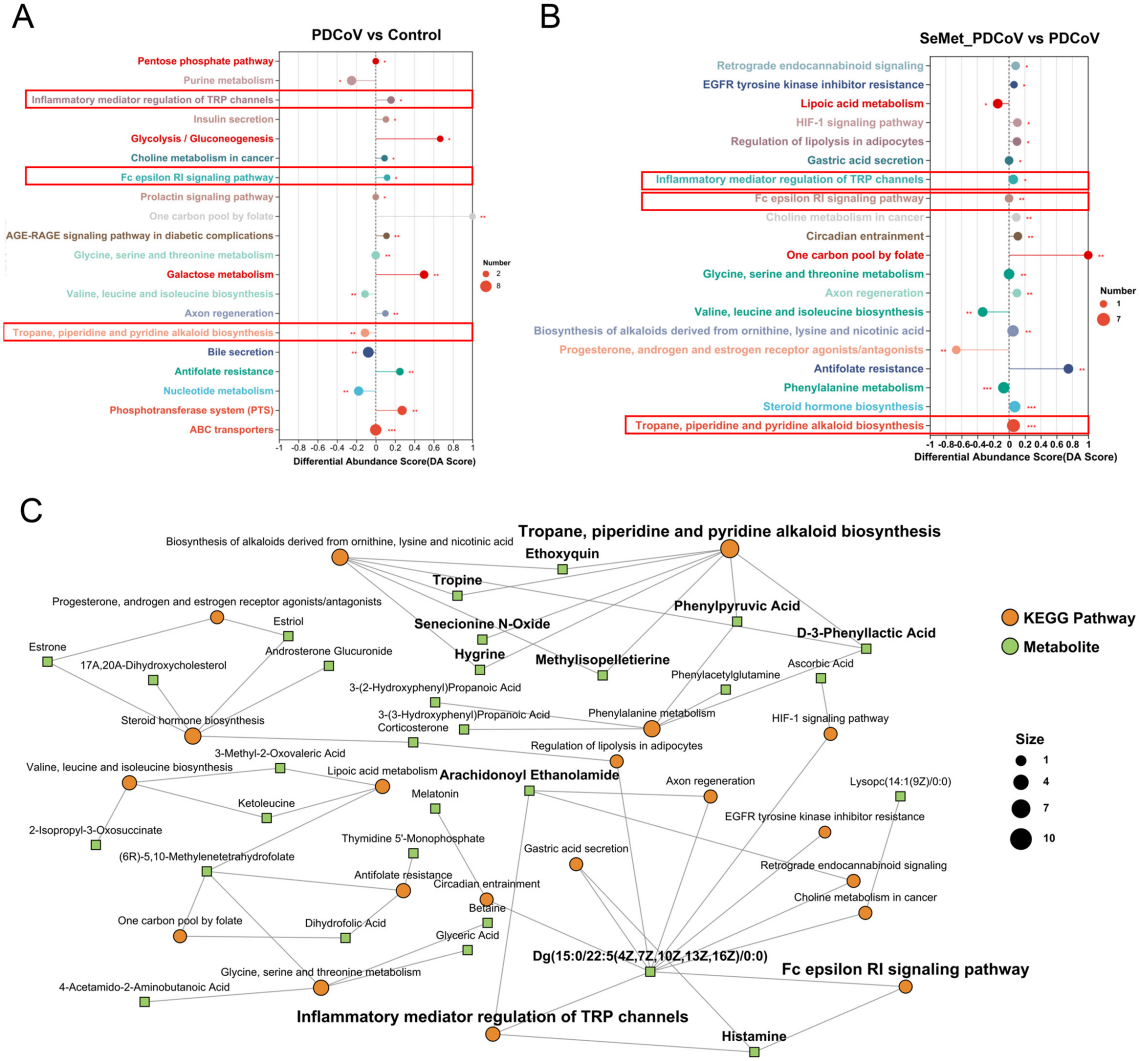
The effects of SeMet on the pathway enrichment in PDCoV-infected mice. **(A)** Potential metabolic pathways analysis based on significantly different metabolites in the colonic contents of PDCoV and Control group mice. **(B)** Potential metabolic pathways analysis based on significantly different metabolites in the colonic contents of SeMet_PDCoV and PDCoV group mice. **(C)** KEGG enrichment analysis network diagram. In **(A, B)**, differential abundance (DA) score values converging to 1 or −1 indicate an upward or downward trend in the expression of all annotated differential metabolites in the pathway. The lengths of the line segments represent the absolute value of the DA score. The size of the dots indicates the number of annotated differential metabolites in the pathway. In **(C)**, the green square nodes indicate metabolites; the orange dots indicate KEGG pathways, and the larger dots represent the higher number of metabolites in the pathway (*n*= 6).

MetaboAnalyst (Xia and Wishart, 2011) was used to link the 541 differential metabolites to potential relevant pathways. MetaboAnalyst 3.0 was then used to identify the impact value of these pathways. As can be seen in Figure 7A, PDCoV infection significantly altered some metabolic pathways. SeMet supplementation modulated the changes in metabolic pathways induced by PDCoV, bringing three of these pathways closer to normal levels, including inflammatory mediator regulation of TRP channels, Fc epsilon Rl signaling pathway, and Tropane, piperidine and pyridine alkaloid biosynthesis (Figures 7A, B). Additionally, Figure 7C showed the disturbed metabolic pathways and associated metabolites, based on the relationship between three metabolomic signatures.

### 3.3 Correlation analysis between microbiota and metabolomic phenotype

**Figure 8:**
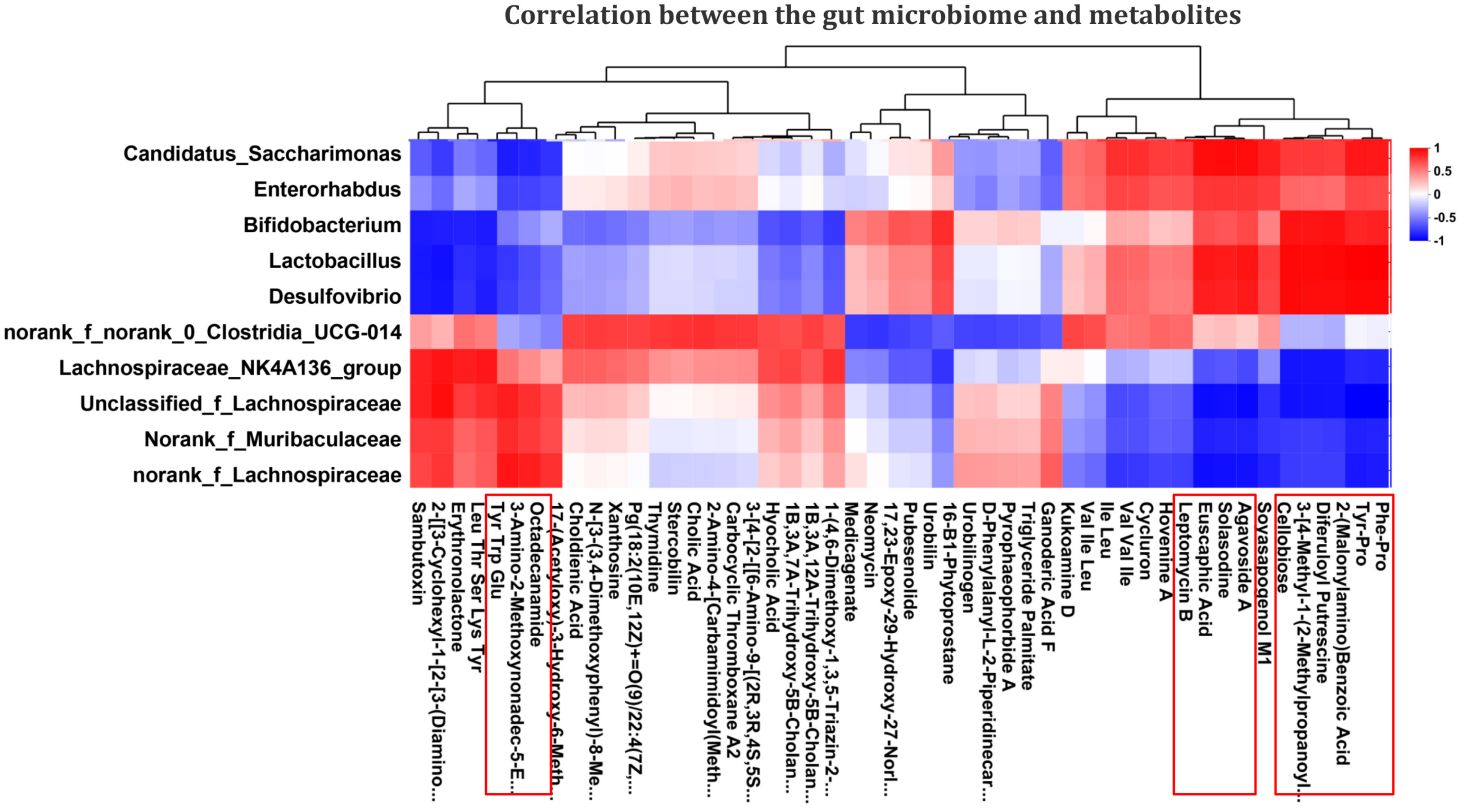
Correlation analysis of the effect of SeMet on intestinal flora and metabolites in PDCoV-infected mice. The left side of the figure shows the genus-level names, and the bottom shows the metabolite names. Each grid in the figure indicates the correlation between two attributes (genus and metabolite association features), and different colors represent the magnitude of the correlation coefficients between the attributes. Red indicates significant positive correlation (*P* < 0.05), blue indicates significant negative correlation (*P* < 0.05), and white indicates that the correlation was not significant (*P* > 0.05) (*n* = 6).

A total of 10 different genera and their relative abundance were determined in the Control, PDCoV, and SeMet + PDCoV groups (Figure 4F). In this work, specific associations between the relative abundance of the identified bacterial g and the top 50 altered metabolites in the different experimental groups were analyzed. As shown in Figure 8, the relative abundance of the genera *Candidatus_Saccharimonas, Enterorhabdus, Bifidobacterium, Lactobacillus*, and *Desulfovibrio* were positively correlated with Phe-Pro. Tyr-Pro, 2-(Malonyl-amino)benzoic acid, 3-[4-methyl-1-(2-methylpropyl)]-1H-indole-2-carboxamide, 3,5-diferuloylputrescine, cellobiose, solasodine, euscaphic acid, agavoside A, and leptomycin B (*P* < 0.05), and negatively correlated with Octadecanamide, 3-amino-2-methoxynonadec-5, and tyrosine, tryptophan and glutamate (*P* < 0.05). Interestingly, the genera *Lachnospiraceae_NK4A136_group, Unclassified_f_Lachnospiraceae, Norank_f_Muribaculaceae*, and *norank_f_Lachnospiraceae* showed negative correlations with octadecanamide, 3-amino-2-methoxynonadec-5-en-4-one, and tyrosine, tryptophan and glutamate (*P* < 0.05) and positive correlations with Phe-Pro, Tyr-Pro, 2-(Malonyl-amino)benzoic acid, 3-[4-methyl-1-(2-methylpropyl)] benzoic acid, difurfurylputrescine, cellobioside, solasodine, euscaphic acid, agavoside A, and leptomycin B (*P* < 0.05). It is worth noting that the genera interacting with metabolites are not entirely consistent with those shown in Figure 4 (Enterorhabdus replaced Akkermansia), possibly because Enterorhabdus has a stronger correlation with metabolites. These correlation data suggested PDCoV-inoculated mice exhibited significant taxonomic perturbations in the intestinal microbiome, which may result in a significantly altered metabolomic profile. SeMet altered the metabolomics of the gut microbes in PDCoV-infected mice.

## 4 Discussion

As a devastating enteropathogenic animal coronavirus, PDCoV could cause intestinal damage in pigs, chickens, and mice, severely affecting their survival (Liang et al., 2019; Li et al., 2020a; Hu et al., 2016; Zhang H. et al., 2022). This leads to significant losses in the livestock industry and economy, as well as posing a serious threat to global public health. Consequently, the search for effective antiviral strategies has become a focal point of public interest. Se, as an important trace element, has attracted attention in recent years for its remarkable antiviral properties. SeMet could alleviate PDCoV-induced intestine injury in piglets and mice, mainly by improving histopathological parameters (Li, 2021; Li et al., 2025). Intestinal health is influenced by lifestyle, diet, and the structure of intestinal microbiota (Moco et al., 2012). Se can interact with gut microbiota, affecting the host's health (Kasaikina et al., 2011; Qiao et al., 2022; Zhang et al., 2021; Zhang H. et al., 2024). In addition, gut microbiota may contribute to the pathogenesis of PDCoV (Zhang J. et al., 2022; Zhang Y. et al., 2024), and the clinical symptoms of PDCoV infection in piglets can be alleviated by regulating intestinal flora (Zhang et al., 2025). In order to explore whether the effects of SeMet improving intestine injury are related to the intestinal microbiota and metabolites, the gut microbiota's diversity and metabolomics in the colon of mice were evaluated. The results showed that SeMet could regulate the intestinal microbiota and metabolites in mice infected with PDCoV. The analysis also revealed a significant correlation between bacterial genera and metabolites.

The intestine is the habitat of a variety of dynamic microbial ecosystems. The diversity of intestinal microbiota is one of the most important elements in resisting the colonization of invading pathogens (Keesing et al., 2010). Studies have shown that PDCoV could significantly reduce the richness and diversity of intestinal microbiota in piglets (Li et al., 2020a). Interestingly, a significant increase in diversity was shown in the PDCoV-infected SPF chickens at 5 dpi (Li et al., 2020b). In this study, there were no significant differences in microbiota diversity among the Control group, the PDCoV group, and the SeMet + PDCoV group. The conflicting results from different studies may be due to differences in species susceptibility to PDCoV. In addition, the microbiota dysbiosis index was higher after PDCoV infection and significantly improved after SeMet supplementation, suggesting that PDCoV infection disrupts the structure of the intestinal flora and that SeMet alleviates PDCoV-induced intestinal dysbiosis.

The normal gut symbionts form a stable community that resists the invasion of pathogens or non-native bacteria and ensures colonization resistance (Freter, 1955; Bohnhoff et al., 1954; Stecher and Hardt, 2008). In terms of species composition (Phylum, Family, and Genus), PDCoV changed the structure of the gut microbiota. Interestingly, the SeMet treatment restored the changes in gut microbiota closer to the control group. At the Phylum level, the main core of the gut microbiota is composed of *Bacteroidetes* and *Firmicutes* (Beheshti-Maal et al., 2021). The change of the Firmicutes/Bacteroidetes ratio may be an important indicator of intestinal ecological disorder (Li et al., 2020a,b), which is usually associated with disease susceptibility (Ley et al., 2006). The results of this study were similar to those of previous research. Interestingly, at the family level, we found that the relative abundance of probiotics (Lachnospiraceae, Muribaculaceae, and Akkermansiaceae) in the PDCoV group had increased, accompanied by a decrease in probiotics (Lactobacillaceae, Bifidobacteriaceae, and norank_o_Clostridia UCG-014). The Lachnospiraceae, as an important member of Firmicutes, exists widely in the human gut and is considered to be potentially beneficial. However, Lachnospiraceae abundance also increases in the intestinal lumen of subjects with different diseases, although the taxa of this family have repeatedly demonstrated their ability to produce beneficial metabolites for their host (Vacca et al., 2020). The Muribaculaceae family is a group of bacteria belonging to the Bacteroidetes order. This family is attached to the mucous layer and shows a strong correlation with inflammatory bowel disease (Lee et al., 2019). Clostridia UCG-014 belongs to the Firmicutes phylum, and its increased abundance helps to restore the gut microbiome's homeostasis, thereby regulating metabolism and alleviating hyperlipidaemia (Duan et al., 2024).

In addition, at the Genus level, there appear to be complex interactions between viral strains and resident bacteria. *Lactobacillus* and *Bifidobacterium*, as the main probiotic genera, were significantly decreased in the PDCoV group. Previous research indicated that piglets infected with PDCoV HNZK-P5 showed a substantial reduction in *lactobacillus* (Zhang J. et al., 2022; Zhang Y. et al., 2024). Probiotics are an important part of the intestinal mucosal barrier, which can effectively resist the penetration of pathogenic microorganisms (Cai et al., 2020), regulate intestinal flora (Cai et al., 2020), produce antiviral metabolites (Al Kassaa et al., 2014), and inhibit the production of pro-inflammatory cytokines (Ghadimi et al., 2012; Hedin et al., 2007). Notably, the abundance of certain probiotics, including *Lachnospiraceae_NK4A136_group* and *Akkermansia*, increased significantly in the PDCoV group. We hypothesize that this may be related to competition between microorganisms. In conclusion, SeMet was found to effectively modulate PDCoV-induced changes in the gut microbiota.

Metabolites are often considered as a bridge between genotype and phenotype, and changes in metabolite levels can directly reveal the function of genes, thus revealing biochemical and molecular mechanisms more effectively (Shen et al., 2023). Changes in metabolic status are caused by gut microbial imbalances in piglets infected with PDCoV (Shi et al., 2024). We hypothesized that the effect of SeMet on the intestinal microbiota in PDCoV-infected mice could extend to impact the intestinal metabolic profile. Metabolic profiling analysis showed that PDCoV infection resulted in metabolic disorders, whereas SeMet treatment significantly mitigated PDCoV-induced metabolic alterations. Amino acids have a variety of important functions in living organisms. They play a role in the synthesis of proteins and other important biomolecules, as well as providing intermediate metabolites for the tricarboxylic acid cycle and gluconeogenesis. Amino acids are involved in the process of viral infection and innate immunity, which is a complex process that involves many different factors (Tomé, 2021). PDCoV infection resulted in changes in the expression patterns of a variety of amino acids (Wang G. et al., 2024). In the present study, we found that the levels of both dipeptides were decreased in the PDCoV group, including Phe-Pro and Tyr-Pro. Research has shown that Phe-Pro is the only *in vivo* active cholesterol-lowering dipeptide among 400 kinds of dipeptides (Banno et al., 2019). PDCoV infection could cause a disturbance in cholesterol metabolism, and further promote its own replication (Zhang J. et al., 2022; Barrantes, 2022). Interestingly, PDCoV infection increased Tyr, Trp and Glu metabolites. This may be to regulate the tricarboxylic acid cycle within the host cell in order to provide amino acids and energy for viral replication (Wang G. et al., 2024). In addition, we also noted an increase in the level of Octadecanamide during PDCoV infection. Fatty amides are well-known mediators of immune response (Lowin et al., 2019; Servettaz et al., 2010). We speculated that the increase in octadecanamide levels was correlated with altered immunity during PDCoV infection.

Pathway enrichment analysis revealed that SeMet supplementation altered the dysregulation of metabolic pathways triggered by PDCoV, including inflammatory mediator regulation of TRP channels, Fc epsilon Rl signaling pathway, and Tropane, piperidine and pyridine alkaloid biosynthesis. The activity of TRPA1 channels and Fc ε [var epsilon] RI signaling has been shown to mediate proinflammatory cytokine responses (Lee et al., 2016; Wu et al., 2021; Péterfy et al., 2008; Li et al., 2021). Supplementation of SeMet down-regulated the inflammatory mediator regulation of TRP channels and Fc epsilon Rl signaling pathways, which may be a cascade reaction of SeMet's inhibition of inflammatory responses. Additionally, SeMet supplementation also up-regulated in Tropane, piperidine and pyridine alkaloid biosynthesis, the content of which had a significant positive correlation with antioxidant activity (Luo et al., 2023). This is consistent with the antioxidant effect of SeMet. Furthermore, this study revealed distinct associations between metabolites and gut microbiota, indicating that perturbations in the gut microbiota are closely related to alterations in the metabolic phenotype.

## 5 Conclusion

This study evaluated the impact of dietary supplementation with SeMet on the gut microbiome and its metabolites in the PDCoV-infected mice. PDCoV infection caused severe dysbiosis of the gut microbiome. SeMet supplementation had a positive impact on regulating the gut microbiome during PDCoV infection. Among these, Lactobacillus and Bifidobacterium may play crucial roles in ameliorating PDCoV-induced intestinal damage. Disturbance to the intestinal microbiota led to significant alterations in the metabolomics profile. SeMet supplementation regulated metabolic alterations induced by PDCoV, including Phe-Pro, Tyr-Pro, Tyr, Trp, Glu, and Octadecanamide. Thereby alleviating the symptoms of PDCoV-induced intestinal injury in mice through multiple mechanisms. These results further elucidate the mechanism of SeMet alleviating intestinal injury in mice infected with PDCoV and provide new strategies to control PDCoV infection. However, the specific bacterial communities or metabolic products involved remain unclear. More detailed isolation and validation are therefore needed to explore the mechanism by which SeMet inhibits PDCoV infection. This will allow more precise research to be conducted on dealing with PDCoV infection in the future.

## Data Availability

The obtained raw sequencing reads have been added to the NCBI SRA page (Accession Number: PRJNA1261576). Other data generated during the study are included in this article.

## References

[B1] Al Kassaa I. Hober D. Hamze M. Chihib N. E. Drider D. (2014). Antiviral potential of lactic acid bacteria and their bacteriocins. Probiotics Antimicrob. Proteins 6, 177–185. 10.1007/s12602-014-9162-6 24880436

[B2] Banno A. Wang J. Okada K. Mori R. Mijiti M. Nagaoka S.. (2019). Identification of a novel cholesterol-lowering dipeptide, phenylalanine-proline (FP), and its down-regulation of intestinal ABCA1 in hypercholesterolemic rats and Caco-2 cells. Sci. Rep. 9:19416. 10.1038/s41598-019-56031-8 31857643 PMC6923426

[B3] Barrantes F. J. (2022). The constellation of cholesterol-dependent processes associated with SARS-CoV-2 infection. Prog. Lipid Res. 87:101166. 10.1016/j.plipres.2022.101166 35513161 PMC9059347

[B4] Beheshti-Maal A. Shahrokh S. Ansari S. Mirsamadi E. S. Yadegar A. Mirjalali H.. (2021). Gut mycobiome: the probable determinative role of fungi in IBD patients. Mycoses 64, 468–476. 10.1111/myc.13238 33421192

[B5] Bohnhoff M. Drake B. L. Miller C. P. (1954). Effect of streptomycin on susceptibility of intestinal tract to experimental Salmonella infection. Proc. Soc. Exp. Biol. Med. Soc. Exp. Biol. Med. 86, 132–137. 10.3181/00379727-86-21030 13177610

[B6] Brown A. J. Won J. J. Graham R. L. Dinnon K. H. 3rd. Sims A.C. Feng J. Y.. (2019). Broad spectrum antiviral remdesivir inhibits human endemic and zoonotic deltacoronaviruses with a highly divergent RNA dependent RNA polymerase. Antiviral Res. 169, 104541. 10.1016/j.antiviral.2019.104541 31233808 PMC6699884

[B7] Cai R. Cheng C. Chen J. Xu X. Ding C. Gu B.. (2020). Interactions of commensal and pathogenic microorganisms with the mucus layer in the colon. Gut Microbes 11, 680–690. 10.1080/19490976.2020.1735606 32223365 PMC7524288

[B8] Duan Y. Guo F. Li C. Xiang D. Gong M. Yi H.. (2024). Aqueous extract of fermented Eucommia ulmoides leaves alleviates hyperlipidemia by maintaining gut homeostasis and modulating metabolism in high-fat diet fed rats. Phytomed. Int. J. Phytother. Phytopharmacol. 128:155291. 10.1016/j.phymed.2023.155291 38518640

[B9] Ferreira R. L. U. Sena-Evangelista K. C. M. de Azevedo E. P. Pinheiro F. I. Cobucci R. N. Pedrosa L. F. C.. (2021). Selenium in human health and gut microflora: bioavailability of selenocompounds and relationship with diseases. Front. Nutr. 8:685317. 10.3389/fnut.2021.685317 34150830 PMC8211732

[B10] Freter R. (1955). The fatal enteric cholera infection in the guinea pig, achieved by inhibition of normal enteric flora. J. Infect. Dis. 97, 57–65. 10.1093/infdis/97.1.57 13242854

[B11] Ghadimi D. Helwig U. Schrezenmeir J. Heller K. J. de Vrese M. (2012). Epigenetic imprinting by commensal probiotics inhibits the IL-23/IL-17 axis in an in vitro model of the intestinal mucosal immune system. J. Leukoc. Biol. 92, 895–911. 10.1189/jlb.0611286 22730546

[B12] Hedin C. Whelan K. Lindsay J. O. (2007). Evidence for the use of probiotics and prebiotics in inflammatory bowel disease: a review of clinical trials. Proc. Nutr. Soc. 66, 307–315. 10.1017/S0029665107005563 17637082

[B13] Hu H. Jung K. Vlasova A. N. Saif L. J. (2016). Experimental infection of gnotobiotic pigs with the cell-culture-adapted porcine deltacoronavirus strain OH-FD22. Arch. Virol. 161, 1–14. 10.1007/s00705-016-3056-8 27619798 PMC7087098

[B14] Kasaikina M. V. Kravtsova M. A. Lee B. C. Seravalli J. Peterson D. A. Walter J.. (2011). Dietary selenium affects host selenoproteome expression by influencing the gut microbiota. FASEB J. 25, 2492–2499. 10.1096/fj.11-181990 21493887 PMC3114522

[B15] Keesing F. Belden L. K. Daszak P. Dobson A. Harvell C. D. Holt R. D.. (2010). Impacts of biodiversity on the emergence and transmission of infectious diseases. Nature 468:647. 10.1038/nature09575 21124449 PMC7094913

[B16] Lednicky J. A. Tagliamonte M. S. White S. K. Elbadry M. A. Alam M. M. Stephenson C. J.. (2021). Independent infections of porcine deltacoronavirus among Haitian children. Nature 600, 133–137. 10.1038/s41586-021-04111-z 34789872 PMC8636265

[B17] Lee K. I. Lee H. T. Lin H. C. Tsay H. J. Tsai F. C. Shyue S. K.. (2016). Role of transient receptor potential ankyrin 1 channels in Alzheimer's disease. J. Neuroinflammation 13:92. 10.1186/s12974-016-0557-z 27121378 PMC4847235

[B18] Lee K. S. Palatinszky M. Pereira F. C. Nguyen J. Fernandez V. I. Mueller A. J.. (2019). An automated Raman-based platform for the sorting of live cells by functional properties. Nat. Microbiol. 4, 1035–1048. 10.1038/s41564-019-0394-9 30886359

[B19] Ley R. Turnbaugh P. Klein S. Gordon J. (2006). Microbial ecology: human gut microbes associated with obesity. Nature 444, 1022–1023. 10.1038/4441022a 17183309

[B20] Li H. Y. (2021). Study on the effects and mechanism of selenomethionine against porcine deltacoronavirus infection. Henan Agricultural University, Zhengzhou, Henan, China.

[B21] Li H. Y. Li B. X. Liang Q. Q. Jin X. H. Tang L. Ding Q. W.. (2020a). Porcine deltacoronavirus infection alters bacterial communities in the colon and feces of neonatal piglets. MicrobiologyOpen 9:e1036. 10.1002/mbo3.1036 32239666 PMC7349149

[B22] Li H. Y. Zhang H. L. Zhao F. J. Wang S. Q. Wang Z. X. Wei Z. Y.. (2020b). Modulation of gut microbiota, short-chain fatty acid production, and inflammatory cytokine expression in the cecum of porcine deltacoronavirus-infected chicks. Front. Microbiol. 11:897. 10.3389/fmicb.2020.00897 32582042 PMC7287039

[B23] Li H. Y. Zhang T. J. Guo X. Guo Y. P. (2025). Protective effect and mechanism of selenomethionine on intestinal injury in mice infected with porcine deltacoronavirus. Acta Microbiol. Sin. 65, 4101–4118. 10.13343/j.cnki.wsxb.20250164

[B24] Li J. Liu R. Sun M. Wang J. Wang N. Zhang X.. (2021). The FcεRI signaling pathway is involved in the pathogenesis of lacrimal gland benign lymphoepithelial lesions as shown by transcriptomic analysis. Sci. Rep. 11:21853. 10.1038/s41598-021-01395-z 34750466 PMC8576038

[B25] Li Y. Chen D. Su J. Chen M. Chen T. Jia W.. (2023). Selenium-ruthenium complex blocks H1N1 influenza virus-induced cell damage by activating GPx1/TrxR1. Theranostics 13, 1843–1859. 10.7150/thno.83522 37064873 PMC10091872

[B26] Liang Q. Zhang H. Li B. Ding Q. Wang Y. Gao W.. (2019). Susceptibility of chickens to porcine deltacoronavirus infection. Viruses 11:573. 10.3390/v11060573 31234434 PMC6631122

[B27] Lowin T. Schneider M. Pongratz G. (2019). Joints for joints: cannabinoids in the treatment of rheumatoid arthritis. Curr. Opin. Rheumatol. 31, 271–278. 10.1097/BOR.0000000000000590 30920973

[B28] Luo Z. Liu L. Nie Q. Huang M. Luo C. Sun Y.. (2023). HPLC-based metabolomics of Dendrobium officinale revealing its antioxidant ability. Front. Plant Sci. 14:1060242. 10.3389/fpls.2023.1060242 36760636 PMC9902878

[B29] Moco S. Martin F. P. Rezzi S. (2012). Metabolomics view on gut microbiome modulation by polyphenol-rich foods. J. Proteome Res. 11, 4781–4790. 10.1021/pr300581s 22905879

[B30] Mori H. Maruyama F. Kato H. Toyoda A. Dozono A. Ohtsubo Y.. (2013). Design and experimental application of a novel non-degenerate universal primer set that amplifies prokaryotic 16S rRNA genes with a low possibility to amplify eukaryotic rRNA genes. DNA Res. 21, 217–227. 10.1093/dnares/dst052 24277737 PMC3989492

[B31] Péterfy H. Tóth G. Pecht I. Erdei A. (2008). C3a-derived peptide binds to the type I FcepsilonR and inhibits proximal-coupling signal processes and cytokine secretion by mast cells. Int. Immunol. 20, 1239–1245. 10.1093/intimm/dxn083 18653698

[B32] Qiao L. Dou X. Song X. Chang J. Pi S. Zhang X.. (2022). Protective effect of biogenic selenium nanoparticles against diquat-induced acute toxicity via regulation of gut microbiota and its metabolites. Food Chem. Toxicol. 170:113480. 10.1016/j.fct.2022.113480 36257488

[B33] Ren Z. Jia G. He H. Ding T. Yu Y. Zuo Z.. (2022). Antiviral effect of selenomethionine on porcine deltacoronavirus in pig kidney epithelial cells. Front. Microbiol. 13:846747. 10.3389/fmicb.2022.846747 35242124 PMC8886123

[B34] Roman M. Jitaru P. Barbante C. (2014). Selenium biochemistry and its role for human health. Metallomics Integr. Biometal Sci. 6, 25–54. 10.1039/C3MT00185G 24185753

[B35] Sartori G. Jardim N. S. Marcondes Sari M. H. Dobrachinski F. Pesarico A. P. Rodrigues L. C.. (2016). Antiviral action of diphenyl diselenide on herpes simplex virus 2 infection in female BALB/c Mice. J. Cell Biochem. 117, 1638–1648. 10.1002/jcb.25457 26639776

[B36] Selenius M. Rundlöf A. K. Olm E. Fernandes A. P. Björnstedt M. (2010). Selenium and the selenoprotein thioredoxin reductase in the prevention, treatment and diagnostics of cancer. Antioxid. Redox Signaling 12, 867–880. 10.1089/ars.2009.2884 19769465

[B37] Sepúlveda R. T. Zhang J. Watson R. R. (2002). Selenium supplementation decreases coxsackievirus heart disease during murine AIDS. Cardiovasc. Toxicol. 2, 53–61. 10.1385/CT:2:1:53 12189280

[B38] Servettaz A. Kavian N. Nicco C. Deveaux V. Chéreau C. Wang A.. (2010). Targeting the cannabinoid pathway limits the development of fibrosis and autoimmunity in a mouse model of systemic sclerosis. Am. J. Pathol. 177, 187–196. 10.2353/ajpath.2010.090763 20508030 PMC2893662

[B39] Shen S. Zhan C. Yang C. Fernie A. R. Luo J. (2023). Metabolomics-centered mining of plant metabolic diversity and function: past decade and future perspectives. Mol. Plant 16, 43–63. 10.1016/j.molp.2022.09.007 36114669

[B40] Shi Y. Li B. Cheng J. Tao J. Tang P. Jiao J.. (2024). Microbial community and metabolome analysis of the porcine intestinal damage model induced by the IPEC-J2 cell culture-adapted porcine deltacoronavirus (PDCoV) infection. Microorganisms 12:874. 10.3390/microorganisms12050874 38792704 PMC11124095

[B41] Stecher B. Hardt W. D. (2008). The role of microbiota in infectious disease. Trends Microbiol. 16, 107–114. 10.1016/j.tim.2007.12.008 18280160

[B42] Tomé D. (2021). Amino acid metabolism and signalling pathways: potential targets in the control of infection and immunity. Eur. J. Clin. Nutr. 75, 1319–1327. 10.1038/s41430-021-00943-0 34163018 PMC8220356

[B43] Vacca M. Celano G. Calabrese F. M. Portincasa P. Gobbetti M. de Angelis M.. (2020). The controversial role of human gut *Lachnospiraceae*. Microorganisms 8:573. 10.3390/microorganisms8040573 32326636 PMC7232163

[B44] Wallenberg M. Misra S. Björnstedt M. (2014). Selenium cytotoxicity in cancer. Basic Clin. Pharmacol. Toxicol. 114, 377–386. 10.1111/bcpt.12207 24529300

[B45] Wang C. Chen H. Chen D. Zhao M. Lin Z. Guo M.. (2020). The inhibition of H1N1 influenza virus-induced apoptosis by surface decoration of selenium nanoparticles with β-thujaplicin through reactive oxygen species-mediated AKT and p53 signaling pathways. ACS Omega 5, 30633–30642. 10.1021/acsomega.0c04624 33283112 PMC7711941

[B46] Wang G. Cao Y. Xu C. Zhang S. Huang Y. Zhang S.. (2024). Comprehensive transcriptomic and metabolomic analysis of porcine intestinal epithelial cells after PDCoV infection. Front. Vet. Sci. 11:1359547. 10.3389/fvets.2024.1359547 38855411 PMC11160942

[B47] Wang H. Shi D. Chen Y. Wang Z. Yuan Y. Yue T.. (2024). Dietary supplementation with novel selenium-enriched Pichia kudriavzevii regulates gut microbiota and host metabolism in mice. Food Funct. 15, 10896–10912. 10.1039/D4FO03633F 39417221

[B48] Wu C. K. Wu C. L. Lee T. S. Kou Y. R. Tarng D. C. (2021). Renal tubular epithelial TRPA1 acts as an oxidative stress sensor to mediate ischemia-reperfusion-induced kidney injury through MAPKs/NF-κB signaling. Int. J. Mol. Sci. 22:2309. 10.3390/ijms22052309 33669091 PMC7956664

[B49] Xia J. Wishart D. S. (2011). Web-based inference of biological patterns, functions and pathways from metabolomic data using metaboanalyst. Nat. Protoc. 6, 743–760. 10.1038/nprot.2011.319 21637195

[B50] Zhai X. Wang S. Zhu M. He W. Pan Z. Su S.. (2019). Antiviral effect of lithium chloride and diammonium glycyrrhizinate on porcine deltacoronavirus in vitro. Pathogens 8:144. 10.3390/pathogens8030144 31505777 PMC6789623

[B51] Zhang H. Ding Q. Yuan J. Han F. Wei Z. Hu H.. (2022). Susceptibility to mice and potential evolutionary characteristics of porcine deltacoronavirus. J. Med. Virol. 94, 5723–5738. 10.1002/jmv.28048 35927214

[B52] Zhang H. Hao Z. Zhang R. Tong J. Wang X. Liu J.. (2024). Artemisia argyi polyphenols attenuates DSS-induced colitis in mice by regulating the structural composition of gut microbiota. Phytomed. Int. J. Phytother. Phytopharmacol. 132:155897. 10.1016/j.phymed.2024.155897 39032279

[B53] Zhang J. Yang G. Wang X. Zhu Y. Wang J. (2022). 25-Hydroxycholesterol mediates cholesterol metabolism to restrict porcine deltacoronavirus infection via suppression of transforming growth factor β1. Microbiol. Spectr. 10:e0219822. 10.1128/spectrum.02198-22 36314946 PMC9769798

[B54] Zhang Y. Si L. Gao J. Shu X. Qiu C. Zhang Y.. (2024). Serial passage of PDCoV in cell culture reduces its pathogenicity and its damage of gut microbiota homeostasis in piglets. mSystems 9:e0134623. 10.1128/msystems.01346-23 38349151 PMC10949489

[B55] Zhang Y. Si L. Shu X. Qiu C. Wan X. Li H.. (2025). Gut microbiota contributes to protection against porcine deltacoronavirus infection in piglets by modulating intestinal barrier and microbiome. Microbiome 13:93. 10.1186/s40168-025-02092-z 40189556 PMC11974153

[B56] Zhang Z. X. Xiang H. Sun G. G. Yang Y. H. Chen C. Effect L. i. T.. (2021). of dietary selenium intake on gut microbiota in older population in Enshi region. Genes Environ. 43:56. 10.1186/s41021-021-00220-3 34903302 PMC8667455

